# Hybrid approach to model the spatial regulation of T cell responses

**DOI:** 10.1186/s12865-017-0205-0

**Published:** 2017-06-21

**Authors:** Anass Bouchnita, Gennady Bocharov, Andreas Meyerhans, Vitaly Volpert

**Affiliations:** 1 Institut Camille Jordan, UMR 5208 CNRS, University Lyon 1, Villeurbanne, 69622 France; 2 Laboratoire de Biométrie et Biologie Evolutive (LBBE), UMR 5558 CNRS, University Lyon 1, Villeurbanne, 69622 France; 3 Mohammadia School of Engineering, Mohamed V University, Rabat, 10080 Morocco; 4 Institute of Numerical Mathematics, Russian Academy of Sciences, Gubkina Street 8, Moscow, 119333 Russian Federation; 5 Infection Biology Laboratory, Department of Experimental and Health Sciences, Universitat Pompeu Fabra, Doctor Aiguader, 88, Barcelona, 08003 Spain; 6 ICREA, Pg. Lluís Companys 23, Barcelona, 08010 Spain; 7 INRIA Team Dracula, INRIA Lyon La Doua, Villeurbanne, 69603 France

**Keywords:** Immune system, T cell, Spatial dynamics, Multi-scale regulation, Hybrid model

## Abstract

**Background:**

Moving from the molecular and cellular level to a multi-scale systems understanding of immune responses requires the development of novel approaches to integrate knowledge and data from different biological levels into mechanism-based integrative mathematical models. The aim of our study is to present a methodology for a hybrid modelling of immunological processes in their spatial context.

**Methods:**

A two-level hybrid mathematical model of immune cell migration and interaction integrating cellular and organ levels of regulation for a 2D spatial consideration of idealized secondary lymphoid organs is developed. It considers the population dynamics of antigen-presenting cells, CD4 ^+^ and CD8 ^+^ T lymphocytes in naive-, proliferation- and differentiated states. Cell division is assumed to be asymmetric and regulated by the extracellular concentration of interleukin-2 (IL-2) and type I interferon (IFN), together controlling the balance between proliferation and differentiation. The cytokine dynamics is described by reaction-diffusion PDEs whereas the intracellular regulation is modelled with a system of ODEs.

**Results:**

The mathematical model has been developed, calibrated and numerically implemented to study various scenarios in the regulation of T cell immune responses to infection, in particular the change in the diffusion coefficient of type I IFN as compared to IL-2. We have shown that a hybrid modelling approach provides an efficient tool to describe and analyze the interplay between spatio-temporal processes in the emergence of abnormal immune response dynamics.

**Discussion:**

Virus persistence in humans is often associated with an exhaustion of T lymphocytes. Many factors can contribute to the development of exhaustion. One of them is associated with a shift from a normal clonal expansion pathway to an altered one characterized by an early terminal differentiation of T cells. We propose that an altered T cell differentiation and proliferation sequence can naturally result from a spatial separation of the signaling events delivered via TCR, IL-2 and type I IFN receptors. Indeed, the spatial overlap of the concentration fields of extracellular IL-2 and IFN in lymph nodes changes dynamically due to different migration patterns of APCs and CD4 ^+^ T cells secreting them.

**Conclusions:**

The proposed hybrid mathematical model of the immune response represents a novel analytical tool to examine challenging issues in the spatio-temporal regulation of cell growth and differentiation, in particular the effect of timing and location of activation signals.

**Electronic supplementary material:**

The online version of this article (doi:10.1186/s12865-017-0205-0) contains supplementary material, which is available to authorized users.

## Background

The immune system is regulated by multiple processes at various levels of biological organization including the genetic-, cellular-, tissue-, organ- and the whole organism levels. The resulting structural and functional complexity of the immune system called for a major shift towards information-rich, systems-based approaches in immunological research. High throughput technologies generate vast amounts of data that facilitate dissection of the immunological processes at ever finer resolution. The need to embed immune processes into their spatial context both at the molecular- and cellular level is a hallmark of the systems immunology approach. In fact, there are many examples of how the fate decisions in the immune system depend on the spatial-temporal dynamics of cytokines, e.g. the interleukin-2 (IL-2) [1] and type I interferon (IFN) [2, 3].

Moving from the molecular and cellular level to a multi-scale model requires the development of novel modelling methodologies for an iterative integration of data from different biological levels into mechanism-based modular mathematical models [4–6]. So far, very few mathematical models have been proposed to describe the multi-scale spatial regulation of immune responses in a genuine hybrid manner [7–10]. The major features of the developed models are summarized in Table 1.

**Table 1:** Overview of the hybrid and multiscale approaches to model the spatial dynamics of immune responses

Model	Phenomena	Process considered	Types of equations	State variables
Baldazzi et al. [7]	Immune response to antigen in lymph node (500 hrs)	Clonal expansion, 3D: transport, reaction-diffusion	Agent-based for cells, PDEs for molecules antigen, chemokines	DCs, B-cells, CD4 ^+^ T cells,
Fallahi-Sichani et al. [8]	Immune response in Tuberculosis, Granuloma (200 days)	Clonal expansion, 2D: chemotaxis, cell-to-cell interactions single-cell state regulation	Agent-based for cells, ODEs for cytokines, 2D geometry of lung tissue	Macrophages, CD8 ^+^ T cells, Treg cells, T *γ* cells, M. tuberculosis, TNF *α*, TNFR
Gong et al. [9]	Immune response to antigen in lymph node (550 hrs)	Clonal expansion, 3D: trafficking, cell-to-cell interactions	Agent-based for cells, anatomicaly based 3D geometry of lymph node	3 states for: DCs, CD4 ^+^ T cells, CD8 ^+^ T cells; Locations for HEVs, FRCs
Prokopiou et al. [10]	Early CD8 ^+^ T-cell response in lymph node (136 hrs)	Clonal expansion, intracellular regulation, 3D: migration, reaction-diffusion	CPM for cells, PDEs for extracellular cytokines, ODEs for intracellular factors	APCs, T-cells, IL-2, IL-2R, Tbet, Caspase, Fas (activated, non-activated)

The model developed in this work takes into account: 1) spatial aspects of the immune response in the lymph node by means of cell and concentration distributions, 2) regulation of T lymphocytes in the lymph node including their asymmetric division and their interaction with extracellular cytokine concentrations, 3) the intracellular regulation of T cells depending on IL-2 and type I IFN, 4) the interaction of the tissue level and the whole organism through the infection level.

Conventional models of the immune response are based on ordinary differential equations, and they do not take into account spatial distributions of cells and concentrations in the lymph node. The multi-scale models previously developed (see Table 1) use a similar agent-based cell description. However they do not take into account processes such as asymmetric cell division, their interaction with IFN or the interaction of the tissue level with the organism level which is one of the key features of our multi-scale model.

One of the essential features of our modelling approach is the description of cells as individual objects which can move, divide, differentiate or die by apoptosis. Dead cells are removed from the computation domain. Cells are considered as soft spheres with their motion described by Newton’s second law for their centers. Cell fate is determined by the intracellular regulation. The spatial distribution of cytokines is described by reaction–diffusion equations, while the intracellular regulation and infection dynamics are described by ordinary differential equations conventionally accepted in mathematical immunology. A similar approach was developed and justified in modelling of other physiological systems such as blood diseases and hematopoiesis [11–17]. Here we develop it for an integrative modelling of immunological processes in their spatial context.

## Methods

### Biophysics of the immune response

To formulate the mathematical model, we consider a part of the lymph node, i.e., the T cell zone, which contains various cell types, mainly the antigen presenting cells (APCs) and subsets of T lymphocytes. Naive T cells and some APCs (such as plasmocytoid Dendritic Cells, pDCs) enter the node with blood flow via the High Endothelial Venules (HEVs) whereas effector and/or memory T cells, and mainly DCs and macrophages home to lymph nodes via afferent lymphatic vessels [18, 19]. Following activation with pathogens, APCs acquire a motile state that allows their translocation to the T cell zone of draining lymph node with the afferent lymph flow [20, 21]. Therefore, we assume that the influx of APCs is proportional to the level of infection in the organism. Differentiation of naive T cells into CD4 ^+^ and CD8 ^+^ T cells occurs in the thymus from progenitor T cells [22]. We suppose that they enter lymph nodes already differentiated and that there is a given influx of each cell type.

**Fig. 1:**
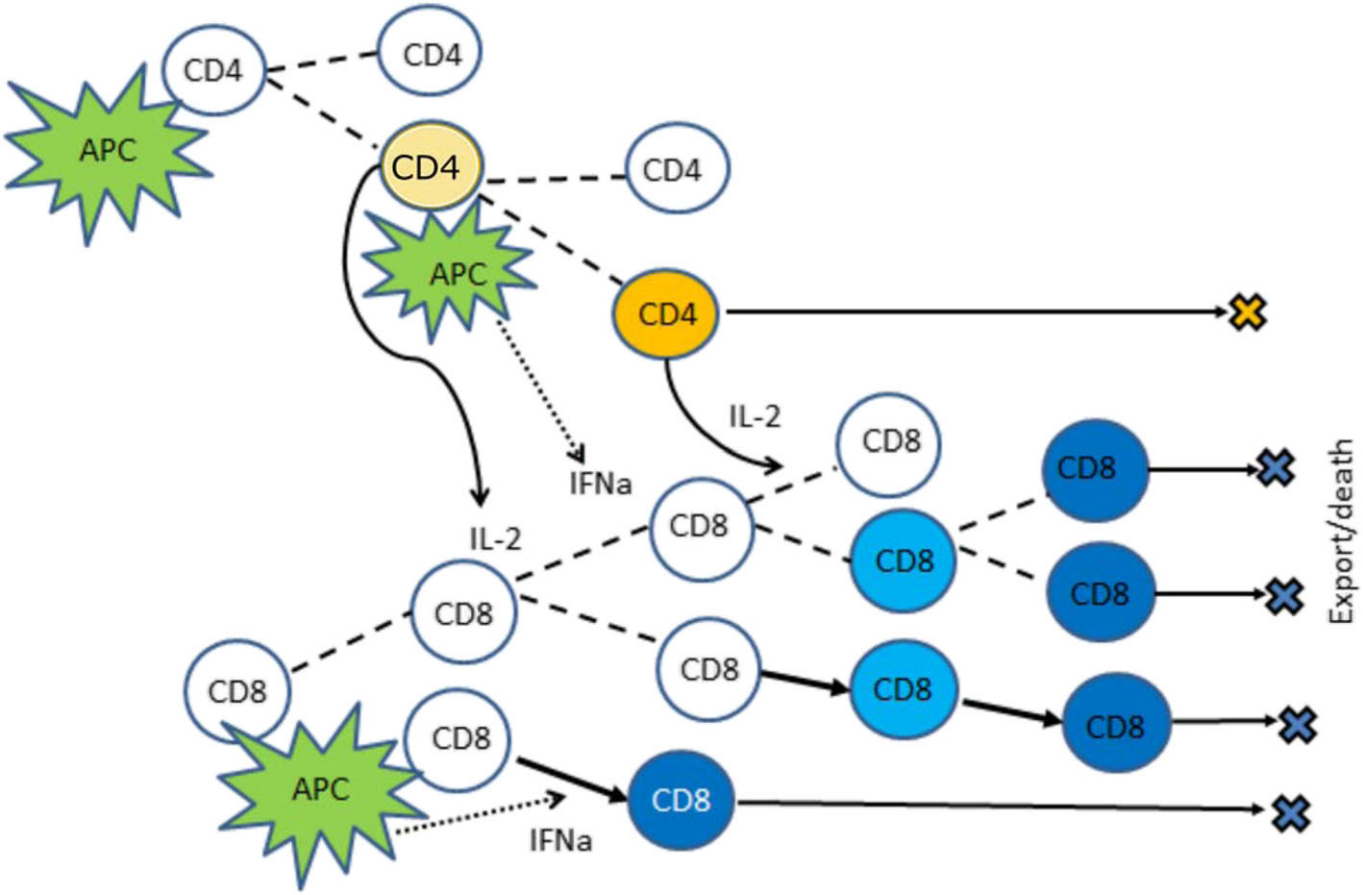
Schematic representation of the model. Naive T cells and antigen presenting cells (APC) enter the lymph node. Due to asymmetric cell division, some T cells differentiate. Mature CD8 ^+^ T cells leave the lymph node and kill infected cells. Mature CD4 ^+^ T cells produce IL-2 that influences cell survival and differentiation. APCs are shown in *green*, naive T cells are *white*. Differentiated CD4 ^+^ T cells are *yellow* and CD8 ^+^ T cells are *blue*. Levels of *yellow* and *blue* indicate cell maturation

**Fig. 2:**
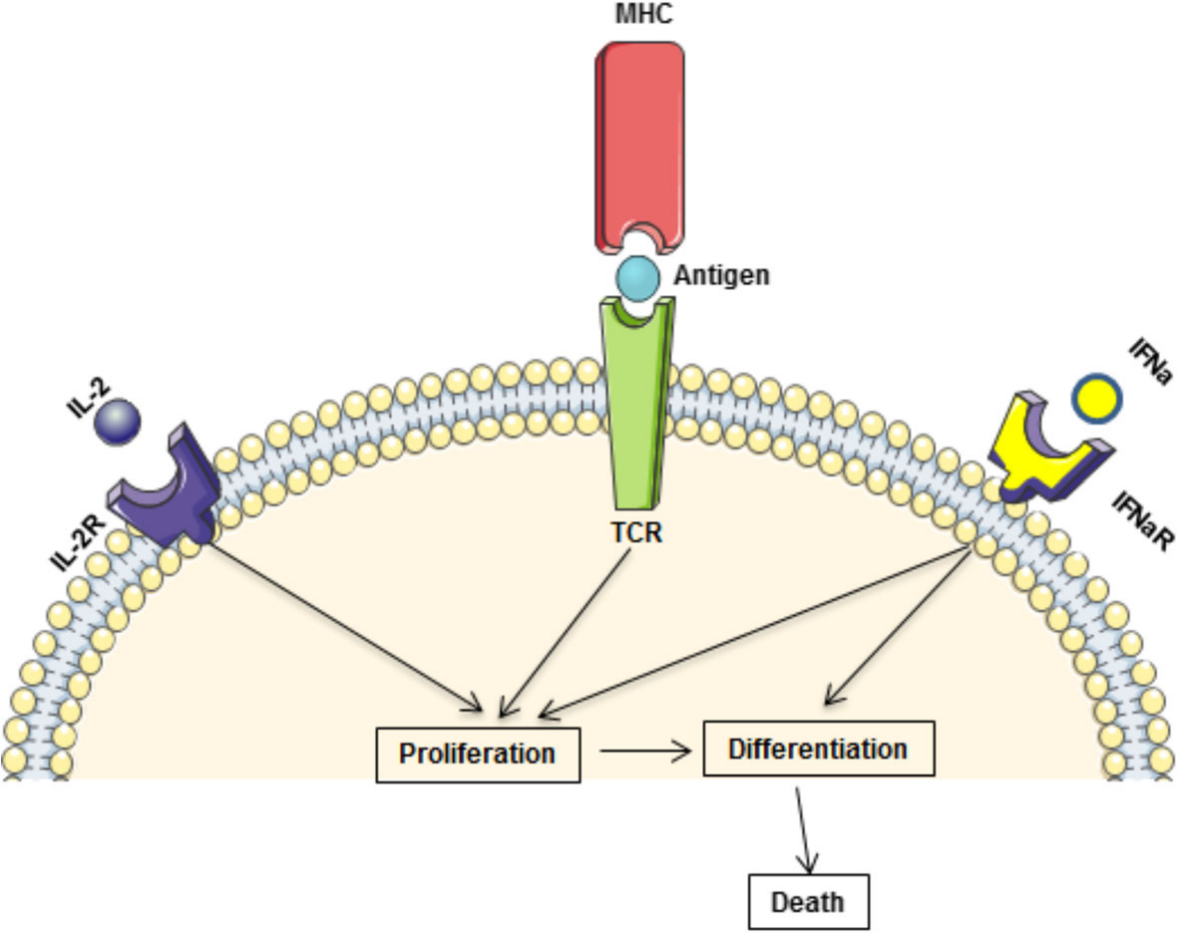
Scheme of the integration of TCR-, type I Interferon- and IL-2 signaling sequence by naïve T cells to adaptively program the balance of growth and differentiation

The APCs bearing foreign antigens activate the clonal expansion of naive T lymphocytes. The activation of T cell division and death is regulated by a set of signals coming from the interactions of the antigen-specific T cell receptors (TCRs) with the MHC class I or class II presented peptides and IL-2 receptors binding IL-2. Naive T cells undergo asymmetric division [23] (Fig. 1). Some of the daughter cells continue to proliferate and differentiate. Mature CD4 ^+^ T cells produce IL-2 [22, 24, 25] which influences survival and differentiation of both CD4 ^+^ and CD8 ^+^ T cells. The proliferation of CD8 ^+^ T cells is stimulated by IL-2 [24]. They can expand their number many thousand-fold. In addition to IL-2 enhancing the proliferation of T cells, APCs start to secrete type I IFN which has an antiviral- and immunomodulatory function. In fact, the effect of IFN *α* depends on the temporal sequence of the signals obtained by naïve T cells [2]. It can change from a normal activation of T cells followed by their proliferation and differentiation to an already differentiated state followed by apoptosis as shown schematically in Fig. 2. Overall, the regulated death of T cells by apoptosis depends on the availability and the timing of TCR, IL-2 and IFN signalling.

Mature CD8 ^+^ T cells (effector cells) leave the lymph node and kill infected cells. Therefore, there is a negative feedback between production of mature CD8 ^+^ T cells and the influx of APCs.

**Fig. 3:**
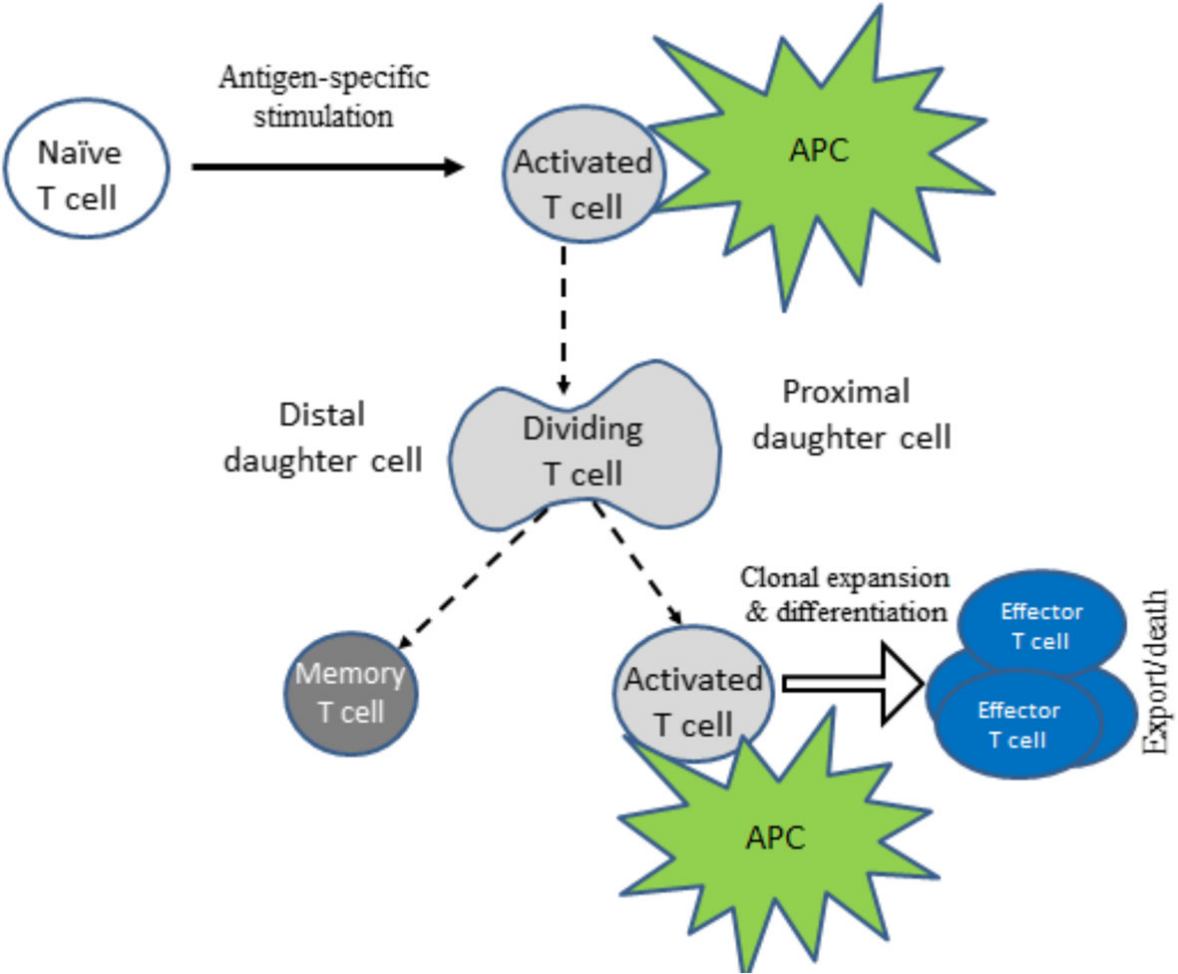
Scheme of the spatial regulation of the asymmetric T cell division in lymph nodes (elaborated from [23])

In the model, an asymmetric T cell division is considered as shown in Fig. 3. Naive T cell entering the draining lymph node is recruited into the immune response after the contact interaction via the T cell receptor (TCR) with APC presenting the foreign antigen. The activation and prolonged contact with APC can results in polarity of the lymphocyte. The position of the contact with the APC determines the direction of cell division and the difference between the daughter cells in terms of their differentiation state. According to [23], the proximal daughter cell will undergo clonal proliferation and differentiation resulting in the generation of terminally differentiated effector cells (mature CD8 ^+^ T cells) that leave the lymph node for peripheral tissues to search and kill infected cells. The distal daughter cell becomes a memory cell. The memory cells are capable of self-renewal by slowly dividing symmetrically in the absence of recurrent infection.

### Hybrid model of cell dynamics

In our model of cell dynamics, cells are considered as individual objects that can move, divide, differentiate and die. Their behavior is determined by the surrounding cells, by intracellular regulatory networks described by ordinary differential equations and by various substances in the extracellular matrix whose concentrations are described by partial differential equations. This approach was used to model hematopoiesis and blood diseases [11–17].

**Cells and concentrations.** Cells in the lymph node:

1. *n*_*APC*_(**x**,*t*) - the density of APCs in T cell zone;
2. *n*_*CD*4_(**x**,*t*) - the density of CD4 ^+^ T cells in T cell zone (with different levels of maturity);
3. *n*_*CD*8_(**x**,*t*) - the density of CD8 ^+^ T cells in T cell zone (with different levels of maturity);

Extracellular variables:

4. *I*_*e*_(**x**,*t*) - the concentration of IL-2 in T cell zone;
5. *C*_*e*_(**x**,*t*) - the concentration of type I IFN in T cell zone;

Intracellular variables:

6. *I*_*i*_(*t*) - the intracellular concentration of IL-2-induced signalling molecules in the *i*th cell;
7. *C*_*i*_(*t*) - the intracellular concentration of type I IFN-induced signalling molecules in the *i*th cell;

The state variables at the level of the whole organism:

8. *N*_*ef*_(*t*) - the total number of effector CD8 ^+^T cells in the body;
9. *N*_*inf*_(*t*) - the total number of infected cells in the body;

**Cell displacement.** In the model, cells are represented by individual elastic spheres. There are two mechanisms of motion of cells in the lymph node. First of all they move in a random way. This motion allows naive T cells to meet APCs which is necessary for their activation, division and differentiation. Second, each two cells, when they meet, they push each other due to a direct mechanical interaction. We consider this interaction as an elastic force acting on cells and influencing their motion. Let us describe it in more detail.

The cells divide and can increase their number which involves pushing each other leading to their displacement in the lymph node. We describe cells displacement by the following model. Let us denote the center of two cells by *x*_1_ and *x*_2_ and their radii by *r*_1_ and *r*_2_ respectively. Then, if the distance *h*_12_ between the two cells is less than the sum of their radii (*r*_1_+*r*_2_), there will be a repulsive force *f*_12_ between them. This force should depend on the difference between (*r*_1_+*r*_2_) and *h*_12_. Let us consider the case of one cell interacting with different cells in the lymph node. The total force applied to this cell will be $F_{i} = \sum _{j\ne i} f_{ij}$. We describe the motion of the particles as the motion of their centers which can be found by the applying Newton’s second law:

1 $$ m \ddot x_{i} + m \mu \dot x_{i} - \sum_{j \neq i} f_{ij} = 0,  $$

where *m* is the mass of the particle, *μ* is the friction factor due to contact with the surrounding medium. The potential force between two cells is given explicitly by:

$$f_{ij} = \left\{ \begin{array}{ccc} K \frac{h_{0} - h_{ij}}{h_{ij} - (h_{0}-h_{1})} &,& h_{0}-h_{1} < h_{ij} < h_{0} \\ 0 &,& h_{ij} \geq h_{0} \end{array} \right., $$

where *h*_*ij*_ is the distance between the centers of the two cells *i* and *j*, *h*_0_ is the sum of their radii, *K* is a positive parameter and *h*_1_ is the sum of the incompressible part of each cell. The force between the particles tends to infinity if *h*_*ij*_ decreases to *h*_0_−*h*_1_.

**Cell division and differentiation.** APC and naive T cells enter the computational domain with a given frequency if there is available space. Naive T cells move in the computational domain randomly. If they contact APC, they divide asymmetrically (Fig. 3). The distant daughter cell is similar to the mother cell, the proximal daughter cell becomes differentiated.

When the cell reaches the half of its life cycle, it will increase its size. When it divides, two daughter cells appear, the direction of the axis connecting their centers is chosen randomly from 0 to 2*π*. The duration of the cell cycle is 18 hours with a random perturbation of −3 to 3 hours.

We consider two levels of maturity of CD4 ^+^ T cells and three levels of CD8 ^+^ T cells. If a differentiated cell has enough IL-2 (see the next paragraph), then it divides and gives two more mature cells. Finally differentiated cells leave the lymph node. In the simulations, this means that they are removed from the computational domain.

**Intracellular regulation.** The survival and differentiation of activated CD4 ^+^- and CD8 ^+^ T lymphocytes depends on the amount of signalling via the IL-2 receptor and the type I IFN receptor. It is controlled primarily by the concentration of the above cytokines in the close proximity of the respective receptors. The signalling events lead to the up-regulation of the genes responsible for cell proliferation, differentiation and death. One can use similar type of equation to model qualitatively the accumulation of the respective intracellular signalling molecules linked to IL-2- and type I IFN receptors. The IL-2 dependent regulatory signal dynamics in individual cells can be described by the following equation:

2 $$ \frac{{dI}_{i}}{dt} = \frac{\alpha_{1}}{n_{T}} I_{e}({\mathbf{x_{i}}},t) - d_{1} I_{i}.  $$

Here *I*_*i*_ is the intracellular concentration of signalling molecules accumulated as a consequence of IL-2 signals transmitted through transmembrane receptor IL2R downstream the signaling pathway to control the gene expression in the *i*th cell. The concentrations inside two different cells are in general different from each other. The first term in the right-hand side of this equation shows the cumulative effect of IL-2 signalling. The extracellular concentration *I*_*e*_ is taken at the coordinate *x*_*i*_ of the center of the cell. The second term describes the degradation of IL-2-induced signalling molecules inside the cell. Furthermore, *n*_*T*_ is the number of molecules internalized by T cell receptors.

In a similar way, the IFN-dependent regulatory signal dynamics in individual cells can be described by the following equation:

3 $$ \frac{{dC}_{i}}{dt} = \frac{\alpha_{2}}{n_{T}} C_{e}({\mathbf{x_{i}}},t) - d_{2} C_{i}.  $$

Here *C*_*i*_ is the intracellular concentration of signalling molecules accumulated as a consequence of IFN signals transmitted through transmembrane receptor IFNR downstream the signaling pathway to control the gene expression in the *i*-th cell. The concentrations inside two different cells are in general different from each other. The first term in the right-hand side of this equation shows the cumulative effect of IFN signalling. The extracellular concentration *C*_*e*_ is taken at the coordinate *x*_*i*_ of the center of the cell. The second term describes the degradation of IFN-induced signalling molecules inside the cell.

To model the fate regulation of growth versus differentiation of the activated cells in relation to the timing of the IL-2 and type I IFN signalling we implement the following decision mechanism.

1. If the concentration of activation signals induced by type I IFN, *C*_*i*_, is greater than some critical level $C_{i}^{*}$ at the beginning of the cell cycle and that of *I*_*i*_, is smaller than the critical level $I_{i}^{*}$, then the cell will differentiate resulting in a mature cell.
2. If the concentration of activation signals induced by IL-2, *I*_*i*_, is greater than some critical level $I_{i}^{*}$ at the end of the cell cycle, then the cell will divide producing two more mature cells.
3. If $C_{i}<C_{i}^{*}$ at the beginning of cell cycle and $I_{i}<I_{i}^{*}$ at the end of cell cycle, then the cell will die by apoptosis and will be removed from the computational domain.

**Stochastic aspects of the model.**

As it is discussed above, mechanical interaction of cells results in their displacement described by equation (1) for their centers. In order to describe random motion of cells we add random variables to the cell velocity in the horizontal and vertical directions.

Duration of cell cycle is given as a random variable in the interval [ *T*−*τ*,*T*+*τ*].

**Extracellular dynamics of cytokines.**

Proliferation and differentiation of T cells in the lymph node depends on the concentration of IL-2 and type I IFN. These cytokines are produced by mature CD4 ^+^ T cells and antigen-presenting cells, respectively. Their spatial distribution is described by a similar reaction-diffusion equation as follows

4 $$ \frac{\partial I_{e}}{\partial t} = D_{IL} \Delta I_{e} + W_{IL} - b_{1} I_{e}.  $$

Here *I*_*ex*_ is the extracellular concentration of IL-2, *D* is the diffusion coefficient, *W*_*IL*_ is the rate of its production by CD4 ^+^ T cells, and the last term in the right-hand side of this equation describes its consumption and degradation. The production rate *W*_*IL*_ is determined by mature CD4 ^+^ T cells. We consider each such cell as a source term with a constant production rate *ρ*_*IL*_ at the area of the cell. Let us note that we do not take into account explicitly consumption of IL-2 by immature cells in order not to introduce an additional parameter. Implicitly this consumption is taken into account in the degradation term.

For type I IFN, the equation and the terms in it have a similar interpretation:

5 $$ \frac{\partial C_{e}}{\partial t} = D_{IFN} \Delta C_{e} + W_{IFN} - b_{2} C_{e}.  $$

Initial and boundary conditions for both concentrations IL-2 and IFN are taken zero. As before, the production rate *W*_*IFN*_ equals *ρ*_*IFN*_ at the area filled by APC cells and zero otherwise.

**Infection.** Mature T cells leave the lymph node. The level of CD8 ^+^ T cells (effector cells) *N*_*ef*_ in the body is determined by the equation

6 $$ \frac{d N_{ef}}{dt} = k_{1} T - k_{2} N_{ef},  $$

where *T* is their number in the lymph nodes. So the first term in the right-hand side of this equation describes production of effector cells in the lymph nodes and the second term their death in the body.

Denote by *N*_*inf*_ the number virus-infected cells. We will describe it by the equation

7 $$ \frac{d N_{inf}}{dt} = f(N_{inf}) - k_{3} N_{ef} N_{inf}.  $$

The first term in the right-hand side of this equation describes growth of the number of infected cells and the second term their elimination by the effector cells. The function *f* will be considered in the form:

$$ f(N_{inf}) = \frac{a N_{inf}}{1 + h N_{inf}} \;, $$

where *a* and *h* are some positive constants.

Finally, the influx of APC cells into the lymph nodes is proportional to the number of infected cells *N*_*inf*_.

This influx is limited by the place available in the lymph node. If there is a free place sufficient to put a cell, the new cells are added. Let us also note that the lymph nodes can increase due to infection in order to produce more effector cells.

## Results

We illustrate the model performance by considering two scenarios, reflecting different spatial patterns of IL-2 and type IFN concentration fields. In the first one, both cytokines have the same diffusion coefficient *D*_*IL*2_=*D*_*IFN*_, whereas in the second case the diffusion rate of IFN is 10-fold faster. The details of the numerical implementation of the hybrid model and the parameter values used for the simulations are presented in Additional file 1: Appendix. Cell population densities and cytokine concentrations are scaled with respect to some reference values. These are determined by the cell density in the lymph node ∼10^5^−10^6^ *mm* ^−3^, the relative proportions of APCs, CD4 ^+^ T cells and CD8 ^+^ T cells [26–32] and the production rate of the cytokines (described in detail in the appendix, see Additional file 1). The considered cell numbers correspond to a computational domain in the T cell zone of about 100*μ* *m*×100*μ* *m*×100*μ* *m*.

The model presented above contains two compartments, lymph node where effector cells are produced and the body where infection develops. The lymph node is described with the hybrid model while infection development in the organism by ordinary differential equations for infected cells and for effector cells. These two compartments are coupled by means of flux of effector cells from the lymph node to the body and by the flux of APC cells to the lymph node.

**Fig. 4:**
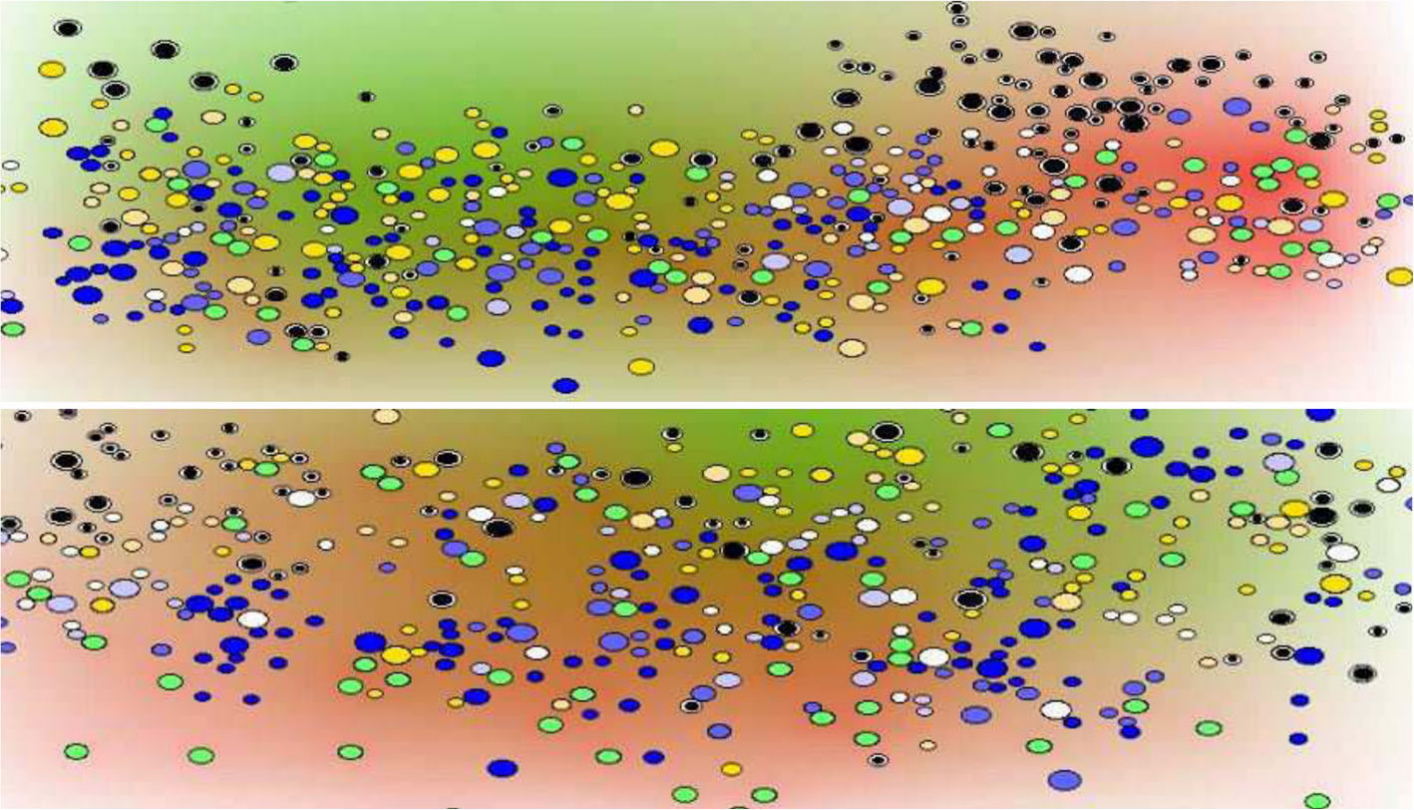
Snapshot of numerical simulations of the cells and cytokines distribution in lymph node. Different cells are shown: APC (*green*), naive CD4 ^+^ T cells (*black*), naive CD8 ^+^ T cells (*white*), three maturity levels of differentiated CD8 ^+^ T cells (*blue*), two maturity levels of CD4 ^+^ T cells (*yellow*). Mature CD4 ^+^ T cells produce IL-2 whose concentration in the extracellular matrix is shown by the level of *green*. APC produce IFN (*red*). The upper figure shows the simulation (day 8 post infection) with equal diffusion coefficients of IL-2 and IFN, in the lower figure (day 80 post infection) the diffusion coefficient of IFN is 10 times larger than the diffusion coefficient of IL-2

**Fig. 5:**
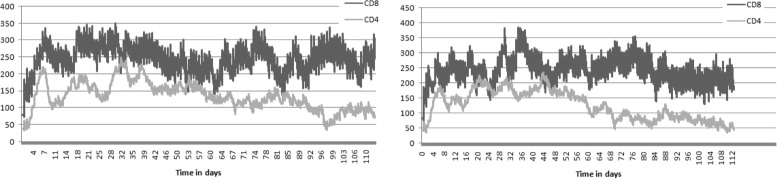
The numbers of CD4 ^+^ and CD8 ^+^ T cells in time in the case of equal diffusion coefficients (*left panel*) and for the diffusion coefficient of IFN 10 times larger than the diffusion coefficient of IL-2 (*right panel*)

**Fig. 6:**
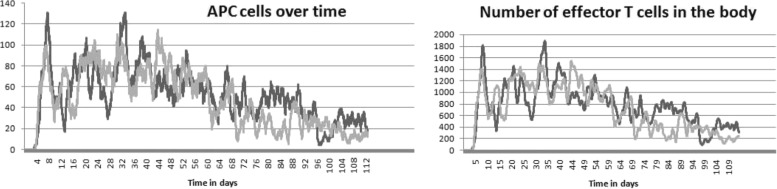
The numbers of APC cells (*left panel*) and effector T cells (*right panel*) in time in the case of equal diffusion coefficients (*black curve*) and for the diffusion coefficient of IFN 10 times larger than the diffusion coefficient of IL-2 (*grey curve*)

**Fig. 7:**
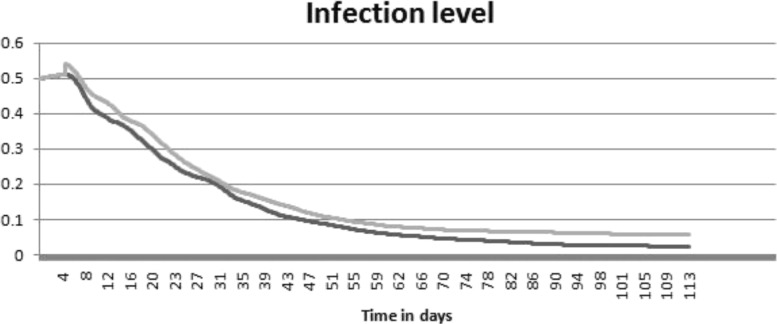
The level of virus infection in the body in the case of equal diffusion coefficients (*black curve*) and for the diffusion coefficient of IFN 10 times larger than the diffusion coefficient of IL-2 (*grey curve*)

The results of the simulations are shown in Figs. 4, 5, 6 and 7. Figure 4 represents a snapshot of the lymph node T cell zone with all cells participating in the simulations: APC cells, naive T cells, differentiated CD4 ^+^ T and CD8 ^+^ T cells. Naive T cells divide when they are close to APC cells. It is an asymmetric division where a proximal daughter cell differentiates while a distant cell remains undifferentiated. Differentiated cells continue their division and maturation in the presence of IL-2 produced by mature CD4 ^+^ T cells [33]. If the level of IL-2 is not sufficient, they die by apoptosis. Mature T cells leave the lymph node. One can see that the cytokine fields are non-uniform and their distribution patterns change essentially if the turnover parameters, e.g. the diffusion coefficient, are varied. Note that the cell distribution is more uniform in the case of large diffusion coefficient of IFN (Fig. 4, lower image) compared with the case of small diffusion coefficient (upper image).

The evolution of the total number of CD4 ^+^ and CD8 ^+^ T cells in the lymph node T cell zone is shown in Fig. 5. The dynamics of APC cells in the lymph node T cell zone and the effector cells in the body is shown in Fig. 6. The magnitude of the immune response is not sufficient to eradicate completely the infection. Indeed, the number of infected cells decreases but remains positive (Fig. 7). As virus infection is not cleared, the cell populations fluctuate around some constant values. Overall, the model reproduces the qualitative patterns of long-term persistent infection (experimental infections and in humans) dynamics (e.g., [34–38]). The primary clonal expansion takes about seven days and is followed by an enhanced long-term T cell response to the persistent infection. The increase in the spread of type I IFN changes the relative distributions pattern of IL-2 and IFN, so that the resulting alteration in the cytokine signalling reduces the clonal expansion and increases the overall level of the virus infection.

**The cumulative numbers of CD4** ^**+**^ **and CD8** ^**+**^ **T cells and virus infection load.** As single simulation runs of the stochastic model are characterized by a fluctuating and overlapping dynamics, we quantified integrative characteristics of the model behavior. To describe the effect of the diffusion coefficient *D*_*IFN*_ on the T-cells production, we compared the cumulative numbers of CD4 ^+^ and CD8 ^+^ T cells as well as the infection load over the overall time of the simulation for the two scenarios. We also show the cumulative numbers of effector T-cells in the body *N*_*ef*_. The results are shown in Table 2.

**Table 2:** Cumulative numbers of key variables of the model over 113 days post infection

Model variable	*D*_*IFN*_=*D*_*IL*_	*D*_*IFN*_=10*D*_*IL*_
Number of CD4 ^+^ T cells	27544	27040
Number of CD8 ^+^ T cells	15194	14139
Number of APCs	4749	5293
The infection load	16.98	19.31
Number of *N*_*ef*_	87849	80967

The net effect of the increase in the diffusion rate of type I IFN is a reduction in the clonal expansion of the T cells, in particular the effector T cells in the peripheral organs (by ∼10*%*) and a rise in the infection level (by ∼20*%*). The changes in the clonal T cell expansion are the consequence of the differences in the cytokine concentration fields, which in turn alter the timing and the sequence of the IL-2 and type I IFN signalling.

**Fig. 8:**
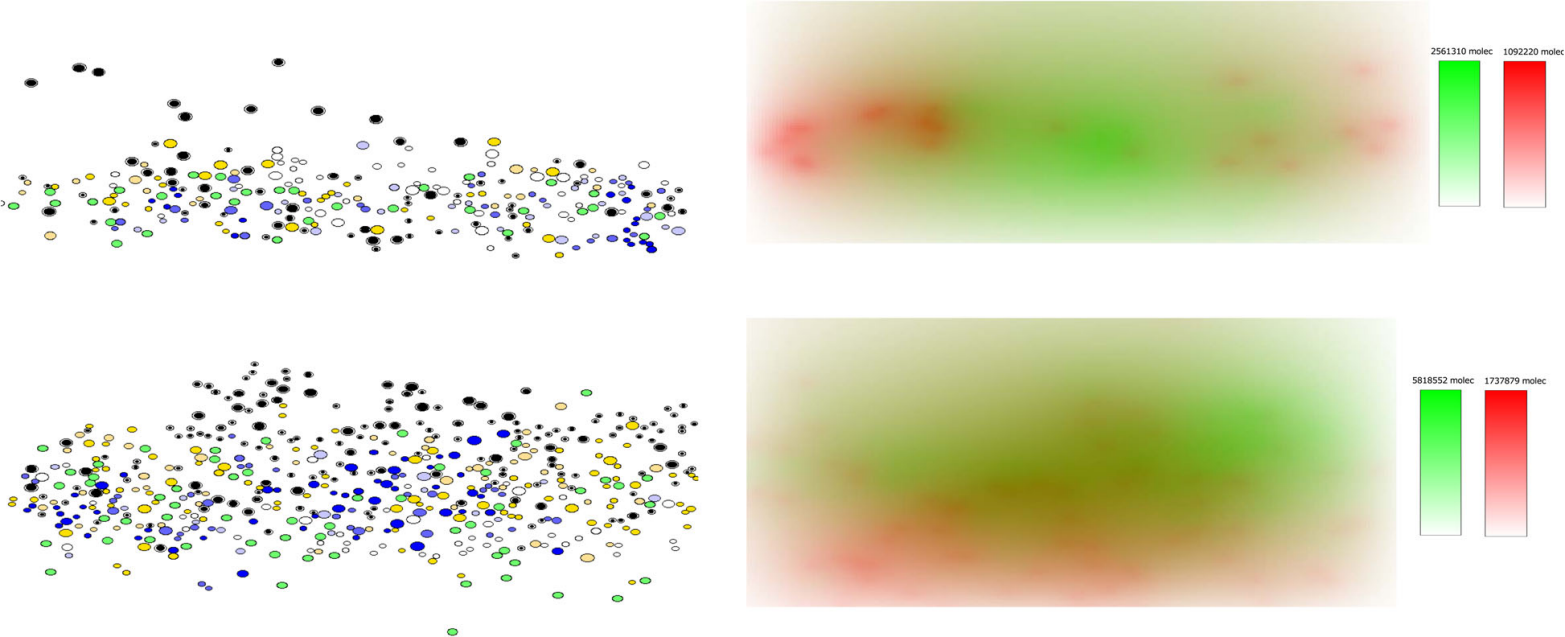
Snapshot of numerical simulations of cell (*left panel*) and cytokine (*right panel*) distribution in a lymph node. First row shows the simulation run (day 4 post infection) with equal diffusion coefficients for IL-2 and IFN, and the lower row represents the outcome of the simulation (day 40 post-infection) with the diffusion coefficient for IFN 10 times larger than the diffusion coefficient for IL-2. APC (*green*), naive CD4 ^+^ T cells (*black*), naive CD8 ^+^ T cells (*white*), three maturity levels of differentiated CD8 ^+^ T cells (*blue*), two maturity levels of CD4 ^+^ T cells (*yellow*). The color bars indicate the cytokine concentration relative to the their maximal value

The types and the relative densities of immune cells considered in the model essentially correspond to the clonal, APC-induced expansion of T cells activated by virus infections (see, e.g., [39]). The motility of T cells in the lymph node is determined by their random motion and mechanical cell-cell interactions [40]. The spatial distribution of cytokines considered in the model (IL-2 and type I IFN), though it requires more detailed investigation, corresponds to the actual understanding of the role of these cytokines. In order to reveal their impact on cell distribution, we show in Fig. 8 the spatial cell distributions and the concentrations of cytokines separately. We can clearly see the role of the cytokine diffusivity on their distributions and on the cell distributions.

## Discussion

The activation of T cell division and death is regulated by a set of signals coming from the interactions of TCRs with the MHC class I and II presented peptides, IFN and IL-2 receptors. However, the effect of type I interferon depends on the temporal sequence of the signals obtained by naïve T cells [2]. It can change from a normal activation of T cells followed by their proliferation and differentiation to an already differentiated state followed by apoptosis. We propose that the altered T cell differentiation and proliferation sequence can result from a spatial separation of the signaling events obtained by T cells, i.e. the TCR versus IFN receptor signaling, due to a generally different location of the APCs and the type I IFN concentration field. The hypothesis is formulated using the results of our study of the spatio-temporal dynamics of the T cell response to infection with the developed hybrid mathematical model integrating intracellular, and systemic levels of the immune response regulation.

Virus persistence in humans is often associated with an exhaustion of T lymphocytes. Many factors can contribute to the development of exhaustion. One of them is associated with a shift from a normal clonal expansion pathway to an altered one characterized by an early terminal differentiation of T cells. The proposed hybrid model allows us to investigate the integrative effects of numerous biophysical and biochemical parameters on the immune regulation that are beyond the scope of the existing experimental techniques.

The need for development of multi-scale integrative models in mathematical immunology is well realized [7, 9, 10]. However, implementation of such models embedded into spatial context of immune responses remains a challenge [4, 41]. Our hybrid model has been developed in a modular form to describe a range of specific interactions and regulations of the immune response. This implies that the structure and composition of the model can be adaptively changed to meet the needs of any specific studies. The present study is based upon a simplified description of the virus-target cell interaction. This block of the hybrid model can be easily refined using the existing set of lumped mathematical models. We presented the results of single runs for two different scenarios. Obviously, multiple runs will be used to address the effect of local and global variations in the parameters on the immune response dynamics in future studies.

The presented methodology for developing a hybrid model of immune processes enables integration of data and knowledge across multiple scales. This is essential for understanding the control of the lower level processes by the properties of the higher level processes. We considered here an important example of the regulation of the T cell fate [2] by the spatial structure and material properties of lymphoid organs in which the transport and cell interaction take place [4].

Mathematical modelling of human immune system represents an important challenge. Whereas a macroscopic level population dynamics of the immune response can be followed experimentally and captured with mathematical models under the framework of mono- or two/three compartmental modelling based on ODEs, the embedding of dynamics and interactions of various cells into the spatial organization of real lymphoid organs represents a fundamental challenge. The effects of spatial heterogeneity of the cytokine fields and cells localization in the lymphoid organs on quantitative and phenotypic features of the T cell responses are poorly understood as these types of parameters are difficult to examine experimentally. Indeed, to describe the cytokine and cellular interactions one need to integrate in a unified manner a range of processes including the lymph flow, constrained diffusion, chemotaxis and haptotaxis in soft tissues. A complementary problem is the assimilation of the data on intracellular regulation of immune cells’ states resulting from a parallel signaling via cell surface receptors to antigens, cytokines, chemokines, hormones, etc. The complexity of the issue is well exemplified in a recent review [42]. Following the presented modelling approach, we hope to be able to examine the sensitivity of the immune responses at the macroscopic level to parameters of the cell interaction at the microscopic level. This should assist in fine tuning of the offset dynamics of immune responses using a broad spectrum of modern immunomodulatory drugs.

## Conclusions

The proposed hybrid mathematical model of the immune response represents a novel analytical tool to examine challenging issues in the spatio-temporal regulation of cell growth and differentiation, in particular the effect of timing and location of activation signals. It allows us to overcome the limitations of reductionist approach to a single factor analysis of the immune regulation and to proceed to the studies of the structure-function relationships in a genuinely systemic manner. As W.E. Paul stated in [33] “…the behavior of immune cells is highly colored by the cellular/molecular environment in which they exist…It is to the quantitative prediction of the outcome of given perturbations in the immune system that we envisage our mathematical/ modeling colleagues will apply themselves.” In our view, hybrid modelling approach provides the means for a comprehensive analysis and interpretation of content-rich meta-data obtained by a broad range of scale-specific data acquisition techniques, including imaging, flow cytometry, transcriptome sequencing in an anatomically correct and immunologically meaningful way.

The practical implementation of a hybrid approach to multiscale modelling presents a number of challenges ranging from the numerical accuracy and consistency of the different methods being used to compute the system components dynamics on one side to the risk of producing modelling artefacts because of the system complexity and parameter uncertainty on the other side. In fact, one needs to have clear computational methodologies for the development of various mathematical modelling tools including simple single-level resolution phenomenological models, large-scale multi-compartmental models and high-resolution multiscale models. In the end, their appropriateness depends on the questions to study and available data.

## Additional file

Additional file 1 Appendix. Numerical method; numerical implementation; values of parameters [43]. (PDF 38 kb)
