# Supplementary material for: Hybrid approach to model the spatial regulation of T cell responses
Source: BMC Immunol. 2017 Jun 21;18(Suppl 1):29. doi: 10.1186/s12865-017-0205-0 (PMC5499095; doi:10.1186/s12865-017-0205-0)
Supplement: Additional file 1 — Appendix. Numerical method; numerical implementation; values of parameters [43]. (PDF 38 kb) [file 12865_2017_205_MOESM1_ESM.pdf]

Appendix

Numerical method

The T cells zone is considered to be a square domain denoted Ω where the reaction-diffusion equations describing extracellular cytokines concentrations are solved. We apply Dirichlet conditions to the four borders of the square domain (Γ). The numerical implementation of the reaction-diffusion equations was done using the finite difference method. Let us consider the boundary value problem (P) for the extracellular cytokine field I. We numerically solve the problem using the alternating direction implicit method. We briefly recall the procedure to be followed below.

$$(P):\begin{cases} \frac{\partial I}{dt} = D\Delta I + W - \sigma I, & in\ \Omega \\ I(x,y,0) = \phi(x,y), & in\ \Omega \\ I = I_0 & \text{on } \Gamma, \end{cases}$$

where D is the diffusion coefficient, W and σ are the production and degradation factors respectively, $\phi(x,y)$ represents the initial condition condition for I, I_0 is a constant prescribed value at the boundaries. We consider the grid $(x_i, y_j, t_n) = (ih, jh, n\delta t)$, where h and δt are the space and time steps respectively. We denote $i = 1, 2, ..., N_x$ and $j = 1, 2, ..., N_y$. To begin with, we rewrite the problem (P) in the following form:

$$(P_1):\begin{cases} \frac{\partial I}{dt} = \frac{D}{2}(\frac{\partial^2 I}{\partial x^2} + \frac{\partial^2 I}{\partial y^2}) + \frac{D}{2}(\frac{\partial^2 I}{\partial x^2} + \frac{\partial^2 I}{\partial y^2}) + W - \sigma I, & \text{in } \Omega \\ I(x,y,0) = \phi(x,y), & \text{in } \Omega \\ I = I_0 & \text{on } \Gamma, \end{cases}$$

We split the first equation of the problem (P_1) into two sub-steps as follows:

$$\begin{cases} \frac{I^{n+1/2}_{i,j} - I^n_{i,j}}{\delta t/2} = D\frac{I^{n+1/2}_{i-1,j} - 2I^{n+1/2}_{i,j} + I^{n+1/2}_{i+1,j}}{h^2} + D\frac{I^n_{i,j-1} - 2I^n_{i,j} + I^n_{i,j+1}}{h^2} + W - \sigma I^{n+1/2}_{i,j}, \\ \frac{I^{n+1}_{i,j} - I^n_{i+1/2,j}}{\delta t/2} = D\frac{I^{n+1}_{i-1,j} - 2I^{n/2}_{i,j} + I^{n+1}_{i+1,j}}{h^2} + D\frac{I^{n+1/2}_{i,j-1} - 2I^{n+1/2}_{i,j} + I^{n+1/2}_{i,j+1}}{h^2} + W - \sigma I^{n+1}_{i,j}. \end{cases} \tag{1}$$

We solve the first equation for each fixed j to obtain $I^{n+1/2}$. Next, we solve the second to obtain I^n. Let us consider the first equation:

$$\frac{I^{n+1/2}_{i,j} - I^n_{i,j}}{\delta t/2} = D\frac{I^{n+1/2}_{i-1,j} - 2I^{n+1/2}_{i,j} + I^{n+1/2}_{i+1,j}}{h^2} + D\frac{I^n_{i,j-1} - 2I^n_{i,j} + I^n_{i,j+1}}{h^2} + W - \sigma I^{n+1/2}_{i,j}.$$

Rearranging the terms we obtain:

$$\underbrace{\frac{D}{h^2}}_{a_{i,j}} I^{n+1/2}_{i-1,j} + \underbrace{\left(-\frac{2D}{h^2} - \frac{1}{\delta t/2} - \sigma\right)}_{b_{i,j}} I^{n+1/2}_{i,j} + \underbrace{\frac{D}{h^2}}_{c_{i,j}} I^{n+1/2}_{i+1,j} =$$

$$\underbrace{-\frac{I^n_{i,j}}{\delta t/2} - D\frac{I^n_{i,j-1} - 2I^n_{i,j} + I^n_{i,j+1}}{h^2} - W}_{f_{i,j}}.$$

Therefore, we can write the first equation of the system 1 in the form:

$$a_{i,j} I_{i-1,j}^{n+1/2} + b_{i,j} I_{i,j}^{n+1/2} + c_{i,j} I_{i+1,j}^{n+1/2} = f_{i,j},$$

for each fixed $j, j = 1, 2, ..., N_y - 1$, we solve numerically:

$$a_i I_{i-1}^{n+1/2} + b_i I_i^{n+1/2} + c_i I_{i+1}^{n+1/2} = f_i, \quad \forall i = 1, 2, ..., N_x - 1 \tag{2}$$

with the boundary conditions $I_{i=0}^n = I_{0,1}$, $I_{i=Nx}^n = I_{0,2}$. We solve the equation (2) using Thomas algorithm. For that, we write the left boundary condition $I_{i=0} = I_{0,1}$as follows:

$$I_0^{n+1/2} = L_{1/2} I_1 + K_{1/2},$$

where $L_{1/2} = 0$ and $K_{1/2} = I_{0,1}$. Then from the equation (2) for $i = 1$:

$$a_1 I_0^{n+1/2} + b_1 I_1^{n+1/2} + c_1 I_2^{n+1/2} = f_1,$$

we obtain $I_1^{n+1/2}$:

$$I_1^{n+1/2} = L_{3/2} I_{1,j} + K_{3/2},$$

where we denote $L_{3/2} = \frac{-c_1}{b_1}$, $K_{3/2} = \frac{a_1 I_{1,0} - f_1}{-b_1}$. We continue this process for $i = 2, 3, ..., N_x - 1$:

$$I_i = L_{i+1/2} I_{i+1} + K_{i+1/2}, \tag{3}$$

where $L_{i+1/2} = \frac{-c_i}{b_i + a_i L_{i-1/2}}$, $K_{i+1/2} = \frac{f_i + a_i K_{i-1/2}}{b_i + a_i L_{i-1/2}}$. We first obtain the coefficients $L_{i+1/2}$, $K_{i+1/2}$ using the formula (2). Next, we find the solution $I^{n+1/2}$ for the sub-step $n+1/2$ by backward sweep using the equation (3). We apply the same procedure on the second equation of the system (1) to compute the next step solution I^n.

Numerical implementation

The source code was written in the Object Oriented Programming (POO) form under C++. The considered time and space units in the model are the minute and the domain length (L) respectively. The library wxWidgets was used to implement a user-friendly interface and visualize the simulations in real-time. The CPU time of numerical simulations was 3 – 4 hours on a computer with four cores and 6GB of RAM.

Values of parameters

The number of T cells in the computational domain is $\sim 3 \times 10^3$ with the proportions of $CD4^+$ and $CD8^+$ T cells being 2 : 1, and the number of APCs ranging from 30 to 300 cells.

For APC, T-cells and infected cells models, the parameters were fitted with experimental data:

1. k_1 The rate of T-cells production and release into the body: 1.8 $/hr$;
2. k_2 The death rate of T-cells in the body: 0.12 $/hr$.

3. k_3 The elimination rate of the infected cells by T-cells 1.8×10^{-6} $/hr$:
4. a A growth rate parameter of infected cells : 0.00024 $/hr$.
5. h A parameter in the growth function of infected cells: 0.006 $/hr$.

For the IL-2 controlled processes the following parameter values were used taken from [43]:

1. n_T - the number of IL-2 molecules internalized by T cells via IL-2 receptors: 2000-5000 per T cell;
2. I_i^* - the saturation concentration of IL-2 for T cell division in vitro: 6×10^{10} molec/ml for 5×10^4 cells/ml;
3. ρ_{IL2} - the secretion rate of IL-2 by single $CD4^+$ T cell: 7×10^5 $molec/hr$;
4. b_1 - the degradation rate of extracellular IL-2: 0.5 $1/hr$;

For illustrative purpose, we assume that the degradation of the IL-2 effect on the intracellular activation d_{inIL2} is equal to $d_{T_{act}}$.

For the type I IFN controlled processes the following parameter values were used taken from [29]:

1. ρ_{IFN} - the secretion rate of type I IFN by single activated APC (plasmacytoid dendritic cell): 1.6×10^4 $molec/hr$;
2. b_2 - the degradation rate of extracellular type I IFN: 0.012 $1/hr$;
3. D_{IL}, D_{IFN} - the diffusion coefficients of IL-2 and type I IFN. As the molecular weights of IL-2 and type I IFN are close to that of myoglobin, we used the following estimate of the diffusion coefficient 0.16 mm^3/h.

The quantitative specification of the effect of signalling on gene activation requires a separate study. To illustrate the model performance, it was enough to assume some reference values as follows: $\alpha_1 \sim \alpha_2 \sim 1$ $1/(molec\ hr)$ and $d_1 \sim d_2 \sim 0.1$ $1/(hr)$ The proliferation and differentiation thresholds have been arbitrarily set to be $I_i^* = 100\ Units(U)$ and $C_i^* = 2000\ U$.
